# Finding Biomarker Signatures in Pooled Sample Designs: A Simulation Framework for Methodological Comparisons

**DOI:** 10.1155/2010/318573

**Published:** 2010-07-04

**Authors:** Anna Telaar, Gerd Nürnberg, Dirk Repsilber

**Affiliations:** Genetics and Biometry, Leibniz Institute for Farm Animal Biology, Wilhelm-Stahl-Allee 2, D-18196 Dummerstorf, Germany

## Abstract

Detection of discriminating patterns in gene expression data can be accomplished by using various methods of statistical learning. It has been proposed that sample pooling in this context would have negative effects; however, pooling cannot always be avoided. We propose a simulation framework to explicitly investigate the parameters of patterns, experimental design, noise, and choice of method in order to find out which effects on classification performance are to be expected. We use a two-group classification task and simulated gene expression data with independent differentially expressed genes as well as bivariate linear patterns and the combination of both. Our results show a clear increase of prediction error with pool size. For pooled training sets powered partial least squares discriminant analysis outperforms discriminance analysis, random forests, and support vector machines with linear or radial kernel for two of three simulated scenarios. The proposed simulation approach can be implemented to systematically investigate a number of additional scenarios of practical interest.

## 1. Introduction

Detection of discriminating patterns in gene expression data can be accomplished by using various methods of statistical learning. Such patterns are of interest as candidate biomarkers/biosignatures to classify samples, for example patients eligible for treatment or not. The Biomarkers Definitions Working Group defines a “biological marker” (biomarker) as a characteristic that is objectively measured and evaluated as an indicator of normal biological processes, pathogenic processes, or pharmacological responses to a therapeutic intervention [1]. For clinical management decisions as in drug-development, risk assessment, diagnostic testing, prognostic stratification, and treatment selection towards individualised medicine, there is an urgent need for robust, valid molecular biomarkers to replace invasive or expensive gold standard methods [2]. In the case of so-called molecular biomarkers, the measured features can be gene expression, protein, hormone, or metabolite levels.

Ideally, biomarkers alone or in combination (as biosignatures) allow consistent classification of an individual to a predefined group. Differential regulation of features, however, does not generally qualify such features as good biomarkers—often validation shows poor classification results for features which were found to be significantly differentially regulated (see, e.g., [3]). Given high individual variances in feature levels (e.g., gene expression), combining several single biomarkers to a so-called biomarker signature (or biomarker panel) has been proposed to help [4]. First, such a signature is more robust with respect to outlier results as a single marker. Second, it enables capturing multidimensional predictive patterns which are not manifest on the univariate level. The latter makes biosignatures appropriate for use in high dimensional data, as in the case of molecular profiling or “OMICs” studies. Classification tools have been developed for analysing multiple features to reveal the optimal biosignature for discrimination, as for example discriminance analysis, partial least squares approaches, support vector machines, or random forests [2, 4, 5]. Many of these methods can cope with both massive collinearity of features as well as the “megavariate” nature of these datasets, where the number of variables is mostly larger or much larger than the number of samples under study [6]. There exist numerous examples for using biosignatures from OMICs data of several sorts for diagnostic or prognostic purposes employing various kinds of statistical learning approaches [7–10]. However, it remains a difficult task to find a valid molecular biomarker which leads to a relevant improvement to existing clinical laboratory values [11]. This is especially true for experiments involving cohort studies of healthy participants where the number of differentially regulated features and patterns can be expected to be very small.

In our study we address an additional problem: Often small amounts of RNA per sample make sample pooling necessary to enable microarray screening experiments. However, sample pooling possibly compromises the analysis results, especially regarding detection of biosignatures: An Affymetrix technical note, for example, points out that once RNA samples are mixed, it is impossible to identify outliers or misclassified samples [12]. As a main observation, it was shown that many transcripts identified as significantly changed in individual samples were not identified in the pooled samples. As a consequence it is recommended that researchers use nonpooled (individual) samples in order to identify statistically significant changes in gene expression. Various and more quantitative investigations have shown that sample pooling leads to biases for the estimates of expression levels as well as it generates differences in lists of detected differentially expressed genes [13, 14]. Shih et al. [15] quantitatively investigated statistical implications of sample pooling regarding imperfect averaging (pool bias) and log transformation (log bias). Sadiq and Agranoff [16] set out to experimentally investigate the consistency of potential biomarker detection when individual case or control serum samples are pooled. They foresee significant limitations for the development of proteomic signature patterns, if such studies would be based on pooled samples: On the one hand their data suggest that low abundance proteins, even when represented in a majority of individual samples, may still be lost during pooling. Moreover sample pooling might result in significant limitations for the development of multivariate signature patterns. Also others propose limitations for the usage of pooling designs for biosignature screening experiments: Kerr for example states that pooling is “generally inappropriate” for classification or clustering studies [17]. Allison and coauthors consider pooling as interfering with the ability to accurately access interindividual variation and co-variation [18]. However, sample pooling is not always avoidable. This raises the question of a good choice of method to detect biosignatures in such cases.

In our study we ask for the actual influence of pooling on the possibility to find multivariate discriminating patterns for the classification task and the dependency on differential expression, technical noise, and choice of method. As this question has not been studied using concrete examples, we set out to develop a simulation framework eligible to consider the following questions: how are multivariate patterns altered by pooling, and which methods are sensitive to pooling designs, which are robust?

## 2. Model and Methods

### 2.1. Theoretical Background

We consider a gene expression experiment and denote the expression of gene *g* in sample *i* by *X*_*g*,*i*_. We assume for each *g* ∈ {1,…, *G*} that *X*_*g*,1_,…, *X*_*g*,*n*_ are independent and identically distributed random variables with mean *μ*_*g*_ and variance *σ* ^2^.

Here *n* denotes the number of individuals and *σ* ^2^ represents the biological variance between the subjects. We get the following gene-wise model for the measured value of the gene expression

(1) $$\begin{array}{c} \begin{array}{cc} Y_{g,i} & =X_{g,i}+{ɛ}_{i}, \end{array} \end{array}$$

by accounting technical variation (measurements errors) *ɛ*_*i*_, *ɛ*_*i*_ ~ *N*(0, *σ*_*ɛ*_^2^).

We only consider the case of an ideal pool where each individual contributes equally to the pool; therefore, the expression level of a pool is the average of the individuals which form the pool (For details see subsection Pooling design). If *X*_*g*,*i*_ is normally distributed and the pool size is *m*_*p*_, 1 ≤ *m*_*p*_ ≤ *n*, it follows that the gene expression of a pool *p*  (1 ≤ *p* ≤ *n*/*m*_*p*_) has the distribution *X*_*g*_^*p*^ ~ *N*(*μ*_*g*_, *σ* ^2^/*m*_*p*_). As a measured value of a pool *p* we get

(2) $$\begin{array}{c} \begin{array}{c} Y_{g}^{p}=X_{g}^{p}+{ɛ}_{p}. \end{array} \end{array}$$

Here *ɛ*_*p*_ ~ *N*(0, *σ*_*ɛ*_^2^), as in (1).

We compare two designs, a single sample design and a pooling design. The variance of ${\bar{Y}}_{g}=\left(1/n\right)\sum_{i=1}^{n}Y_{g,i}$ in the single sample design is equal to $\sigma_{{\bar{Y}}_{g,S}}^{2}=\left(1/n\right)\left(\sigma^{2}+\sigma_{ɛ}^{2}\right)$ and in the pooling design we have $\sigma_{{\bar{Y}}_{g,P}}^{2}=\left(1/n_{P}\right)\left(\sigma^{2}/m_{p}+\sigma_{ɛ}^{2}\right)$. Here *n*_*P*_ denotes the number of pools. The variance of ${\bar{Y}}_{g,S}$ depends on the variance components *σ*_*ɛ*_^2^ and *σ* ^2^; the variance of ${\bar{Y}}_{g,P}$ depends also on the pool size (remember in our case that all pools have the same size); see [13]. When we only consider the biological variance, we get the total variance $\sigma_{{\bar{X}}_{g,S}}^{2}=\sigma^{2}/n$ for the single sample design and $\sigma_{{\bar{X}}_{g,P}}^{2}=\left(1/n_{P}\right)\left(\sigma^{2}/m_{p}\right)$ for the pooling design. We get the same total variance in both designs by choosing *n* = *m*_*p*_ *n*_*P*_.

To exemplify multivariate patterns we consider patterns which were built out of two features for two classes, *A* and *B*. A pattern, as we understand it, allows a partion of a feature space into regions belonging to the classes of samples.

### 2.2. Simulating Gene Expression Data

Simulating two designs, a design of a pooled study and a design of a study with single samples, we consider the influence that pooling has on a classification task. Data were generated randomly for two classes *A* and *B*. Our data matrix has 60 rows (corresponding to the subjects) and 1000 columns (corresponding to the features). The 60 rows are partitioned in 30 rows per class. The matrix contains an informative part and a large noninformative part with respect to the classification. In the noninformative part the values are normally distributed random variables with mean eight and variance 0.2^2^.

We investigate three scenarios for the informative data part. In the first scenario we only simulate ten of 1000 genes as independent differentially expressed between the two classes *A* and *B*. For the second scenario we simulate linear dependent genes (details below for the linear pattern). For the third scenario we combine both independent differentially expressed genes and linear patterns.

For the first scenario we simulate ten independent differentially expressed features. This submatrix forms the first ten columns of the simulated 60 samples × 1000 features data matrix. For each of the ten genes we proceed in the following way. The mean *μ*_*A*_ of class *A* is randomly chosen (according to the uniform distribution) from the interval [6,10]. The 30 gene expression values which belong to the subjects of class *A* are *N*(*μ*_*A*_, 0.2^2^)-distributed random samples. The gene expression values of the class *B* individuals are *N*(*μ*_*A*_ + Δ, 0.2^2^)-distributed, where Δ is randomly chosen according to the uniform distribution on the interval [0.1,0.5]. Technical noise is added with the two variance levels 0.2^2^ and 0.4^2^.

For the second scenario we simulate a two-dimensional pattern. The normally distributed *X*_*g*_1_,*i*_^*A*^ and *X*_*g*_1_,*j*_^*B*^ with *i*, *j* = 1,…, 30, both with variance *σ* ^2^ = 0.2^2^ and a mean randomly chosen according to the uniform distribution on the interval [6,10] form the first component of the pattern, which is built without loss of generality of the genes *g*_1_ and *g*_2_. We calculate the values for *X*_*g*_2_,*i*_^*A*^ in the following way:

(3) $$\begin{array}{c} \begin{array}{c} X_{g_{2},i}^{A}=2X_{g_{1},i}^{A},\;\forall i, \end{array} \end{array}$$

and with the consideration of technical noise *ɛ*_*i*_ we get *Y*_*g*_2_,*i*_^*A*^ = 2*X*_*g*_1_,*i*_^*A*^ + *ɛ*_*i*_ for all *i*. By analogy we calculate the values for class *B*

(4) $$\begin{array}{c} \begin{array}{c} X_{g_{2},j}^{B}=2X_{g_{1},j}^{B}+\delta \;\forall j, \end{array} \end{array}$$

and with the consideration of technical noise we get *Y*_*g*_2_,*j*_^*B*^ = 2*X*_*g*_1_,*j*_^*B*^ + *δ* + *ɛ*_*j*_ for all *j*. We use *δ* = 0.4. Again we evaluate two noise levels with variances 0.2^2^ and 0.4^2^. Thus we simulate pairwise linear dependent genes which are illustrated in Figure 3. Separated by a straight line, this pattern has a simple form. Simulating ten patterns, the simulated matrix which is combined with the noninformative matrix consists of 20 columns.

For the third scenario we implement these two scenarios in combination, simulating again ten independent differentially expressed features and ten linear patterns.

We simulated 500 training sets as described above. As test sets, for each repetition, we simulated again a 60 × 1000 data matrix with 30 rows per class under the same conditions. Comparing a single sample design and a pooling design for each scenario by accounting for the two noise levels, we report the prediction errors of five statistical learning methods. Here we understand as prediction error the relative frequency of wrongly classified samples of the test set.

### 2.3. Pooling Design

**Figure 1:**
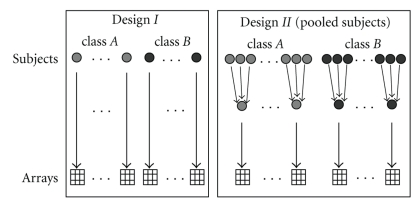
Single sample design and pooling design for class comparison. Design *I* (single samples) and design *I* *I* (pooled samples) for the comparison of class *A* and class *B*. In design *I* one sample is analysed on a single array. In design *I* *I*, at first, pools are built with identical size. Afterwards each pool is hybridized on an array.

Figure 1 shows our two simulated designs for a classification task. The grey circles represent the subjects of class *A* and the black circles the subjects of class *B* (individuals or pools). In the left scheme a single sample is measured on an array. The right scheme shows that pooling takes place before the samples are analysed. Therefore we transform the simulated normally distributed data by exponentiating the data with basis two and pool this transformed data. After the pooling step we transform the data back on the log scale again by taking base-two logarithms of the values. We consider three different pool sizes *m*_*p*_ = 2,3, 5. In each validation step the individuals of a pool were randomly chosen without replacement. For choice of sample size we account for *n* = *m*_*p*_ *n*_*P*_. Every individual sample only contributes to one pool and all original samples were used for our classification task.

In the pooling design the methods are trained with the pooled samples (training set) and tested with independent new single samples (test set).

### 2.4. Methods for Classification

We use five different statistical learning methods.

1. Support vector machines with a linear kernel (SVML). Support vector machine (SVM) classification is based on large-margin separation and kernel functions [19].
2. Support vector machines with a radial basic kernel (SVMR). The radial kernel is an example for a nonlinear classification approach. For both we use the R-package e1071 [20] with a 10-fold cross validation on the training data to assess the prediction error.
3. Random forests: random forests (RF) is an ensemble method which is based on decision trees and makes a classification using majority votes over all trees [21]. We choose the parameters 1000 as number of trees to grow and 20 as number of randomly drawn variables tested for splitting at each node. We use the R-package randomForest.
4. Powered partial least squares discriminant analysis: powered partial least squares discriminant analysis (PPLS-DA) is an advancement of powered partial least squares (PPLS) for highly collinear predictors with Fisher linear discriminant analysis (FLDA). The influence of not so important predictors can be reduced in this approach by restricting the power parameters like in the powered partial least squares (PPLS) approach [22]. The linear combinations of the columns of the predictor matrix that maximize the covariance to the corresponding column of the response matrix build the components (latent variables) which are used for the discriminant analysis. We use the R-package cppls version 2.1–0 with the power parameters (lower = 0, upper = 1); therefore extracted components were only influenced by few features. We also implemented an inner 10-fold cross-validation to choose the number of PPLS components.
5. Linear discriminant analysis: As a univariate method we use the *t*-test and after choosing the ten best candidates with regard to the *P*-value we use these *t*-test candidates for a linear discriminant analysis (LDA).

For the methodological comparison we consider prediction errors of these five methods in a pooled and a single sample design. Confidence intervals are calculated taking a Bootstrap approach [23] as implemented in the R-package varbin.

Our codes are available on request.

## 3. Results

We compare three different scenarios. In the first scenario ten independent genes are simulated with mean value differences between the two classes, in the second scenario ten linear dependent genes discriminating the groups are built, and in the third scenario we combine both.

**Figure 2:**
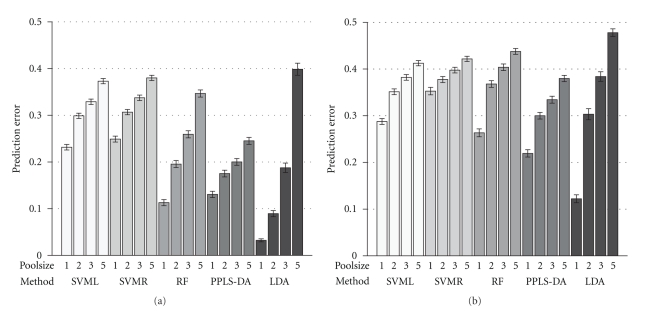
Prediction errors for the first scenario: Ten differentially expressed genes with noise levels of variance 0.2^2^ (a) and 0.4^2^ (b). On the *x*-axis the five statistical methods SVML, SVMR, RF, PPLS-DA and LDA after *t*-test are shown and on the *y*-axis the prediction error is given. The height of a bar specifies the average prediction error of 500 runs for a specific pool size and the 95% bootstrap confidence intervals are shown. The four bars for a method represent the different sizes of pools 1 (single samples), 2,3 and 5 which are indicated by the numbers below the bars.

In the first scenario the difference between the mean values of class *A* and *B* is randomly chosen according to the uniform distribution on the interval [0.1,0.5]. Figure 2 shows the prediction errors of the five methods in the following order: SVML, SVMR, RF, PPLS-DA, and LDA with the ten best *t*-test candidates, and for pools of size *m*_*p*_ = 1,2, 3,5 (*m*_*p*_ = 1 corresponding to individual samples, design *I*) by accounting for two noise levels. For the right panel noise with a variance of 0, 4^2^ (four times biological variance) was added and for the left panel less noise with a variance of 0.2^2^ (same level as the biological variance). The height of the bars represents the mean rate of wrong predicted samples for 500 validation runs, in addition the 95% bootstrap confidence intervals are given. With increasing size of the pools the prediction error increases over all methods. For the single samples (*m*_*p*_ = 1), pools of size two and three, LDA after *t*-test makes the lowest prediction error with around 0.03, 0.09, and 0.19. But the prediction error of LDA almost doubles for each step in size of the pools. Considering PPLS-DA for all sizes of pools the prediction error is under 0.25. The results of the two support vector machine methods are similar with prediction errors between 0.23 and 0.38. For the higher noise level all methods except SVMR show an increase of the prediction errors particularly when comparing the single sample design with a design of pools with size *m*_*p*_ = 2. For both noise levels in the scenario of independent differentially expressed genes LDA shows the highest increase of the error rate while stepping up size of pools. In the case of a pooling design with *m*_*p*_ = 5 and for a noise level of 0.2^2^ the prediction error of LDA is more than 12 times higher as in the single sample design. For a technical noise of 0.4^2^ the LDA prediction error increases nearly by four times between the single sample design and the pooling design with *m*_*p*_ = 5. 

**Figure 3:**
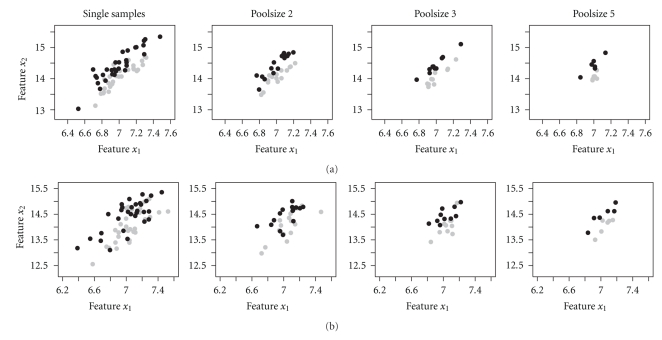
Linear patterns before and after pooling with two noise levels of variance 0.2^2^ (a) and variance 0.4^2^ (b). The grey circles represent the subjects of class *A* and the black circles the subjects of class *B* (30 subjects per class). Pooling effects for the two cases of the linear pattern are shown after pooling randomly two, three and five subjects.

Figure 3 shows the bivariate linear pattern with the two noise levels of variance 0.2^2^ and 0.4^2^ for single samples and pools with the different sizes. The construction of the linear pattern causes also a mean value difference between the two classes of 0.4 for the second gene. The linear pattern is persistent to pooling.

**Figure 4:**
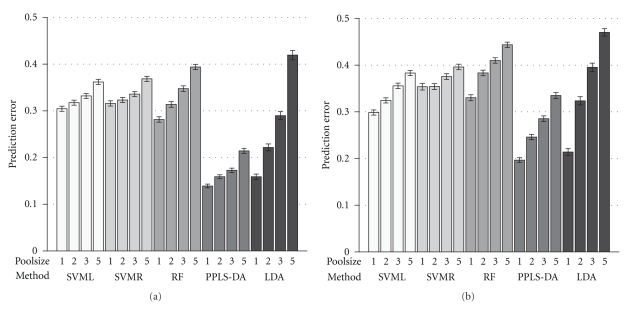
Prediction errors for the second scenario: Ten linear patterns with noise levels of variance 0.2^2^ (a) and 0.4^2^ (b). On the *x*-axis the five statistical methods SVML, SVMR, RF, PPLS-DA and LDA after *t*-test are shown and on the *y*-axis the prediction error is given. The height of a bar specifies the average prediction error of 500 runs for a specific pool size and the 95% bootstrap confidence intervals are shown. The four bars for a method represent the different sizes of pools 1 (single samples), 2, 3 and 5 which are indicated by the numbers below the bars.

In Figure 4 the prediction errors for our second scenario are shown. Again for the right-hand side of this figure a higher noise level was added. Here the prediction error is higher for all methods and pool sizes compared to the first scenario. What is noticeable here is that the two support vector machine methods have a very low increase of prediction error comparing the single sample design with the design of size of pool *m*_*p*_ = 5. Especially SVMR in the higher variance cases has only up to three more misclassifications. Regarding only the right plot of Figure 4, RF shows the highest prediction error for the single samples and all sizes of pools except size *m*_*p*_ = 5. Again LDA reacts with the strongest increase viewed over all pool sizes for both noise levels. PPLS-DA performs in average superior to the other methods for this scenario with prediction errors lower than 0.22 and less than 0.34 for the variance levels similar to the biological variance and four times higher as the biological variance. 

**Figure 5:**
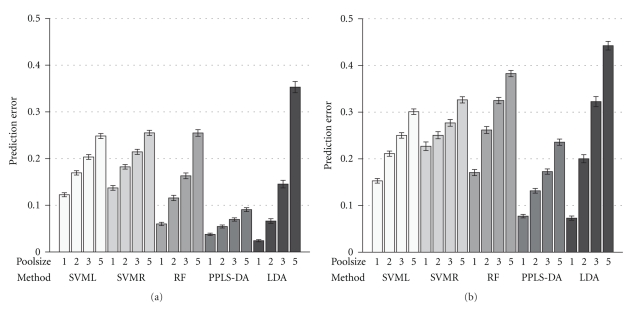
Prediction errors for the third scenario: Ten differentially expressed genes and ten linear patterns with noise levels of variance 0.2^2^ (a) and 0.4^2^ (b). On the *x*-axis the five statistical methods SVML, SVMR, RF, PPLS-DA and LDA after *t*-test are shown and on the *y*-axis the prediction error is given. The height of a bar specifies the average prediction error of 500 runs for a specific pool size and the 95% bootstrap confidence intervals are shown. The four bars for a method represent the different sizes of pools 1 (single samples), 2, 3 and 5 which are indicated by the numbers below the bars.

The result of the third scenario, the combination of independent differentially expressed genes and the linear pattern, is shown in Figure 5. For the different methods the number of wrongly predicted samples is decreasing in comparison to the first and the second scenarios. While increasing the size of the pools PPLS-DA shows a prediction error under 10% for a noise variance of 0.2^2^. Viewed over all settings for this scenario PPLS-DA shows the lowest prediction error. For the different noise levels the prediction error we found behaves very similarly for SVML, SVMR, and RF. 

## 4. Discussion

### 4.1. Summary of Main Results

We propose a simulation framework to explicitly investigate parameters of pattern, pool size, noise, and choice of method and their influence on classification performance. We exemplify our simulation approach by using a two-group classification task and simulated gene expression data with independent genes and linear dependent genes. Our results show a clear increase of prediction error with size of pool when keeping the total number of samples constant, for all five methods investigated. PPLS-DA outperforms the other methods for the linear pattern and the combination of independent differentially expressed genes and the linear pattern for all pooled sample designs (see Figures 4 and 5). We conclude that the proposed simulation approach should be implemented to systematically investigate a number of additional scenarios, patterns and methods of practical interest.

### 4.2. Significance of Results

As often recommended in the context of experimental design eligible for classification studies and, hence, detection of candidate biosignatures, sample pooling should be avoided if possible [12, 17, 18]. For the first time our simulation study illustrates the basis for this recommendation simulating actual illustrative examples. For all methods under comparison a single sample design is clearly preferred in our study—independent from noise levels and pattern scenarios (with independent differentially expressed genes or/and with pairwise linear dependent genes). If pooling is inevitable, only PPLS-DA—for two scenarios of our simulation study (see Figures 4 and 5)—even for the pooling designs provides not very high prediction errors. The support vector machines are most robust against pooling effects in the linear pattern scenario (see Figure 4). Sample pooling is sometimes necessary, as amounts of RNA isolated from single samples may be much less than the required minimum for a microarray gene expression experiment. Also, each tissue isolate can in principle be seen as a pooled sample as the single cells constituting the tissue are most probably not synchronized regarding their regulatory circuit periods. In these cases our results suggest that there are differences between methods of statistical learning regarding this objective. For our examples we chose a bivariate linear pattern as a very simple multivariate pattern. We cannot generalise to all possible patterns and methods. More complex patterns could change the performance of the methods under comparison. In our study we only investigate an example as illustration of our proposed simulation framework.

### 4.3. Constraints and Benefits of Methods

As in our study we wanted to examine pooling effects on a specific pattern, we were not investigating experimental datasets as for example in [24]. Taking a simulation approach we wanted to be able to differentiate if our data showed independent differential gene expression (of single features) or not in order to control if the three described scenarios would have an effect on predictive performance. In addition during simulation we could control the noise level as well as exact type of discriminating pattern present in the data.

When basing biomarker detection on pooled samples, nevertheless the application area of such biomarkers would be the single new sample to be classified, not a pool. In our simulation we take care of this realistic constraint: All test sets consist of single samples while pooling only affects the training sets. Our modelling approach is based on the assumption that after appropriate pretreatment gene expression data (mostly their logs) will be approximately normally distributed [25]. As Zhang et al. [26] point out, other than approached by Shih et al. [15] and Kendziorski et al. [13], pooling occurs on the level of RNA extracts, that is, on the original scale, prior to transformations. Therefore in our simulation approach to pooling, we back-transform the normally distributed single sample data, pool and then log transform again. Moreover in the current implementation of the pooling model there is no equivalent of what Kendziorski et al. [13] call the pooling error, but pools are simulated as mean values of their constituting single samples. Possible influences of realistic pooling errors remain to be investigated.

Independent differentially expressed features were simulated using Δ between 0.1 and 0.5 on the log-scale—which is equivalent to between 1.07- and 1.4-fold changes in gene expression. These values for differential gene expression are comparatively small (compare to example [27]). Our choice was motivated by the typical case for which biomarker detection is not straight forward: no features exist which could be used for discrimination by a simple cutoff-criterion. Instead, information of such candidates has to be combined (using methods of statistical learning) to deliver a good biomarker signature. From our results it is, however, clear that for our choice of independent differential expression and pairwise linear dependent genes all methods under comparison could better use the combination of both which is shown by lower prediction error rates (see Figure 5).

We want to point out that for investigation of pooling effects on classification abilities of different methods, noise regimes and data with independent features with distinction of mean values or with pairwise linear dependent features, it is important to choose a model simple enough to allow investigating the impact of the single effects. It remains an open task to approach possibilities to analyse experimental data in a similar way.

Internal cross-validation loops for the training step were necessary where the methods had additional parameters to optimise [2]. For PPLS, we had to optimize the number of latent variables to be used for the regression. For the support vector machines with the radial kernel we made use of the implemented internal cross-validation to optimise parameter *γ*.

### 4.4. Outlook

It is certainly a demanding future work to fit further realistic simulation scenarios to experimental data and draw conclusions for methodological recommendations for data analysis. However, to date it is unclear how to determine to which extent experimental data consist of univariate differentially expressed genes, and how much is the result of regulatory interactions, that is, multivariate patterns. It would be conceivable to “substract” differentially regulated genes from a dataset to result in a scenario with only multivariate discriminating patterns remaining. Our choice of pattern is largely arbitrary, many more alternative possibilities exist, also truly multivariate patterns where the number of interacting features exceeds two. Hence the pattern dependency of pooling effects remains to be investigated. Similarly our choice of methods is an arbitrary subset from what is currently applied for classification in OMICs datasets. Here we look forward to further implementations of our simulation approach using further methodological alternatives, possibly also for investigating why some methods seem to be sensitive to sample pooling while others are not. Feature selection performance would be a further interesting characteristic—in addition to pure predictive error—to compare different methods using our proposed simulation framework: Does pooling alter the possibility to find the correct, responsible discriminating features?

Summarising we want to propose our approach to investigate effects of sample pooling on classification performances for different datasets (pooling design, patterns, differentially expressed features) and classification methods. As for many occasions pooling cannot be avoided, we look forward to methodological recommendations to analyse such designs with respect to detection of biosignatures for prediction, based on systematic investigations using our proposed framework.
